# Chromosomes as Barcodes: Discovery of a New Species of Black Fly (Diptera: Simuliidae) from California, USA †

**DOI:** 10.3390/insects13100903

**Published:** 2022-10-04

**Authors:** Peter H. Adler, Shaoming Huang

**Affiliations:** 1 Department of Plant and Environmental Sciences, Clemson University, Clemson, SC 29634, USA; 2 San Joaquin County Mosquito & Vector Control District, Stockton, CA 95206, USA

**Keywords:** aquatic insects, *Boreosimulium*, COI barcode, cytogenetics, polytene chromosomes, rivers, *Simulium annulus* species group, taxonomy

## Abstract

**Simple Summary:**

The discovery and elucidation of species, especially those that are structurally similar, often require special tools and approaches. The basic principle of a unique barcode, as used in retail stores and more recently as DNA sequences in taxonomy, is also inherent in the giant banded chromosomes of flies (Diptera). We used the sequence of chromosome bands—the barcode—in the discovery of a new species of black fly from California, USA. The chromosomal barcode indicates this species is unique among all others in the *Simulium* (*Boreosimulium*) *annulus* group to which it belongs. To provide an integrated taxonomic approach, we analyzed and described the larvae, pupae, males, and females of this new species and assigned the formal name *Simulium ustulatum* n. sp. We also provided a traditional DNA barcode using the cytochrome *c* oxidase I (COI) gene. The new species has the most rearranged chromosomes of all North American members of its species group and differs most conspicuously by having a large chromocenter from which all six chromosome arms radiate. The species is structurally unique in the larva and male. The COI barcode enables identification of the female and pupa, which are structurally similar to other species in the group. The new species is the only western North American member of the *Simulium annulus* group that inhabits low-elevation, disturbed landscapes.

**Abstract:**

One of the most popular tools for species discovery and resolution is the DNA barcode, typically based on the cytochrome *c* oxidase I (COI) gene. However, other non-genic barcodes are available for Diptera. The banding sequence of polytene chromosomes in some dipteran cells, particularly of the larval silk glands, can provide a unique species barcode. We used the sequence of bands to reveal a new species of black fly in the *Simulium* (*Boreosimulium*) *annulus* species group from California, USA. To further characterize the species and provide more integrated taxonomy, we morphologically described all life stages above the egg, formally named the species *Simulium ustulatum* n. sp., and provided a conventional COI barcode. The COI barcode confirmed the chromosomal and morphological evidence that the species is a new member of the *S. annulus* group, and enabled identification of the larva and female, which are structurally similar to those of other species. The chromosomal barcode shows that this species has the most rearranged complement, compared with the eight other North American members of its species group, with up to 12 times the number of fixed rearrangements. Up to six chromosomal rearrangements, including autosomal polymorphisms and sex-linked phenomena, are shared with other members of the group. The most unique and conspicuous chromosomal feature of this new species is a large, pale-staining chromocenter from which the six chromosomal arms radiate. The distribution of this univoltine species in lowland rivers of California’s Central Valley could make it vulnerable, given climate change and increasing land development.

## 1. Introduction

The Universal Product Code (barcode), familiar to consumers nearly worldwide, was invented in the early 1970s as a series of vertical lines of varying thickness to identify and track retail products in stores. DNA barcodes gained equivalent familiarity in the biological community with the publication of a seminal paper outlining the methods and utility of a “global bioidentification system” using a DNA sequence from a particular gene, most popularly the cytochrome *c* oxidase I (COI) gene [1]. However, Nature’s physical analogy of the Universal Product Code—a sequence of bands of different thickness—is the set of polytene chromosomes in certain organisms, particularly Diptera and some Collembola. Polytene chromosomes are found in tissues such as salivary glands and ovarian nurse cells that require a high level of product. Once an initial group of cells in these tissues has formed early in larval development, cells cease to divide and begin to increase in size. The chromosomes within each cell, however, continue to duplicate, producing many interphase sister chromatids that remain together as the cell enlarges [2]. A series of dark and light transverse bands characterizes polytene chromosomes, with the dark bands containing more DNA due to dense packing and the light bands expressing the reverse condition.

The pattern of dark and light chromosomal bands is typically species-specific. Chromosomal barcodes, consequently, have been used as taxonomic tools in Diptera, notably the families Chironomidae, Culicidae, Drosophilidae, and Simuliidae [3,4,5,6,7,8]. They have been used to discover and delimit species, infer phylogenies, understand speciation, and map the genome [9,10,11,12,13]. Consequently, polytene chromosomes have been instrumental in targeting the relevant species for controlling vector-borne diseases [14,15,16]. Polytene analyses revealed the African *Simulium damnosum* complex as the largest species complex of arthropod vectors, consisting of more than 50 cryptic species and genetically distinct forms [17].

One of the most extensive bodies of literature on the genetics of natural populations has been developed for the family Simuliidae, which includes about 2400 species worldwide [18]. Field populations of more than 570 species have, to some extent, been studied chromosomally, and the majority of these species have had their polytene chromosomes mapped [19]. The large, distinctly banded polytenes, with a haploid number of two or three, lend themselves to detailed microstructural analysis and are taxonomically essential in a family notorious for its morphological homogeneity [16,20].

The Holarctic subgenus *Simulium* (*Boreosimulium*) is one of about 50 subgenera in the Simuliidae. It consists of three species groups, of which the *S. annulus* group includes 12 nominal species [18]. The immature stages of the group’s members develop early in the year in cold streams and rivers with smooth flow; the females acquire blood from birds [21]. The chromosomes of eight members of the *S. annulus* group have been compared with a standard reference map of the polytene banding sequence [21,22,23].

We present a chromosomal analysis and description of a new species from California, USA—the thirteenth member of the *S. annulus* group—which we discovered through polytene comparisons with all eight nominal species of the group in North America and morphological comparisons with the four additional Palearctic members of the group. The chromosomal barcode of this new species is definitive for identification. We also chromosomally infer related species, provide morphological characters that allow a rapid means of identification, and discuss the novel and shared chromosomal attributes of this new species. Recognizing that chromosomal and morphological approaches have limitations in the discovery and resolution of species, we also use the traditional COI barcode for further diagnostic insight. The COI barcode, while offering numerous taxonomic advantages such as simplicity and applicability to all life stages, similarly has its limitations [24]. We, therefore, discuss the advantages and limitations of the chromosomal and molecular barcodes, and advocate an integrated approach to solving taxonomic problems.

## 2. Materials and Methods

### 2.1. Collection of material

**Figure 1:**
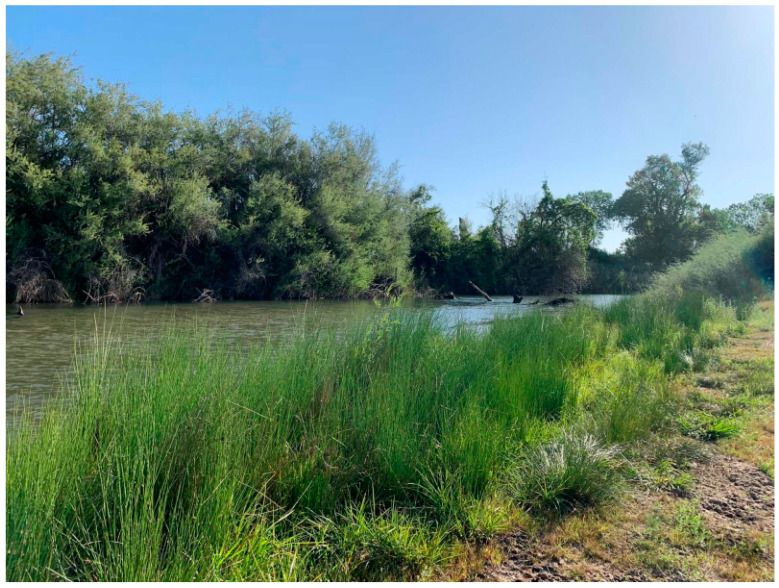
Type locality of *Simulium ustulatum* n. sp., Mokelumne River, San Joaquin County, California, 38°12′36″ N 121°22′32″ W, 4.5 m asl, 8 March 2022.

Material of the new species, including the type series, was collected primarily from the Lower Mokelumne River in San Joaquin County, California (38°12′36″ N 121°22′32″ W), at an elevation of 4.5 m above sea level (asl) (Figure 1). The river originates in the Sierra Nevada range at about 2600 m asl. The Mokelumne River is 153 km long and flows from the central Sierra Nevada into the Central Valley where it empties into the San Joaquin River. It is regulated by the Pardee Dam where water is stored and diverted for municipal use and the Camanche Dam, which regulates releases for irrigation and flood control. The portion of the river downstream from the Camanche Dam is referred to as the Lower Mokelumne River.

The riverbed consists mostly of sand with fine silt and mud along the banks. The water at the sampling site was clear. The predominant submerged aquatic vegetation consisted of Canadian waterweed (*Elodea canadensis*) along the banks and dense masses of the moss *Fontinalis hypnoides* attached to submerged logs. Simuliid larvae and pupae attached to both of these plants. Additional submerged vegetation in the area, but to which simuliids did not attach, included coontail (*Ceratophyllum demersum*) and filamentous algae. The riparian zone consisted mostly of sandbar willow (*Salix exigua*), river bulrush (*Scirpus fluviatilus*), and annual beardgrass (*Polypogon monspeliensis*) along the banks, with valley oak (*Quercus lobata*) and Fremont poplar (*Populus fremontii*) further upland. Canopy cover at the collection site was 0%, with approximately 25–50% canopy cover 50 m upstream. 

The depth, width, and water velocity of the Lower Mokelumne River fluctuate due to controlled reservoir releases upstream. At the location and time of sampling, the Lower Mokelumne River had a maximum depth of about 0.9 m and a width of 17.7 m, with a surface water velocity of 0.49 m/s. The historical average March temperature for the Mokelumne River, according to the California State Water Resources Control Board (upstream, 15.8 km southeast of the sampling site), is 13.1 °C. The pH was 7.2 and the levels of ammonia, nitrite, nitrate, and phosphate were 0 ppm (API Freshwater Master Test Kit, Mars Fishcare North America, Chalfont, PA). Sulfite was in the range of 0–10 mg/L and sulfate < 200 mg/L (Quantofix^®^ colorimetric test strips, Macherey-Nagel GmbH & Co., KG, Düren, Germany).

Larvae and pupae were collected on 8 March and 21 March 2022, respectively, from submerged vegetation attached to woody debris and growing along the riverbanks. One set of larvae was placed in modified Carnoy’s fixative (1 part glacial acetic acid: 3 parts absolute ethanol) and a second set was placed in absolute ethanol. Pupae were reared at room temperature to adults, which were held in darkness for 12–16 h to allow cuticular tanning, and were then fixed with their exuviae in 95% ethanol, along with pupae that failed to produce adults. 

Larvae also were collected on 7 March 2022 from the Stanislaus River in San Joaquin County, 37°42′10″ N 121°10′39″ W, 9 m asl, although in fewer numbers. Like the Mokelumne, the Stanislaus River flows from the Sierra Nevada into the Central Valley where it joins the San Joaquin River and is regulated by several dams and reservoirs for irrigation, domestic water supply, and hydroelectricity.

### 2.2. Chromosomal Procedures and Terminology

Larvae were cut between the fourth and fifth abdominal segments. The posterior portion was opened ventrally with fine needles and treated with Feulgen stain [21]. The stained salivary glands and one gonad for sex determination were dissected out in a drop of tap water on a microscope slide and squashed in a drop of 50% glacial acetic acid under a coverslip [21]. Chromosomes were analyzed under oil immersion and photographed with a Jenoptik ProgRes^®^ SpeedXT Core 5 digital camera mounted on an Olympus BX40 light microscope. Images were imported into Adobe^®^ PhotoShop^®^ Elements 8 to assemble and label chromosome maps. All chromosomal images are from larvae collected at the type locality.

The banding sequences of the long (L) and short (S) arms of each of the three chromosomes (I, II, and III) were compared with the standard reference map for the *S. annulus* species group [22]. *Simulium annulus* (Lundström) was previously referred to as *S. euryadminiculum* Davies until the latter was synonymized with the former [21]. Thus, references to *S. euryadminiculum* or its abbreviation (eu) [22] refer to *S. annulus*. Previously recognized chromosomal rearrangements were named according to their original designations, whereas novel fixed interspecific inversions were numbered beginning after the last-numbered fixed inversion [22]. Polymorphisms were numbered, beginning with ‘1’ in each arm and the prefix ‘us’, representing the first two letters of the new species name, following previous practice [22]. Although this practice for labeling polymorphisms is maintained for the new species, it becomes cumbersome when the inversion is shared among multiple species. For example, IIIL ca, eu, em, Hi-1 of Golini and Rothfels [22] is shared among *S. canonicolum* (Dyar & Shannon), *S. annulus*, *S. emarginatum* Davies, Peterson & Wood, and *S. zephyrus* Adler, Currie & Wood (as ‘Hinton’). Fixed inversions are italicized, but polymorphisms are not. Rearrangements with adequate representation (i.e., cells > 5) were tested for Hardy–Weinberg equilibrium.

### 2.3. Morphological Procedures

Selected specimens were dissected in 80% ethanol for imaging of structures with potential taxonomic value. The larval head capsule, female head, and adult terminalia were transferred to 85% lactic acid and heated in a crucible for ca. 1 min to remove soft tissues. Dissected parts were then transferred to a drop of glycerin in a depression slide, further dissected into component parts if necessary, and oriented for study and imaging. Larval antennae, pupal gills, and adult mouthparts and legs were taken from 80% ethanol, placed in a drop of 50% acetic acid, and positioned under a coverslip for imaging. Cocoons for imaging were placed directly into glycerin.

Structures were photographed at multiple focal planes with a Jenoptik ProgRes^®^ SpeedXT Core 5 digital camera mounted on an Olympus BX40 light microscope. Helicon Focus (version 7.7.5) stacking software was used to form composite images across multiple focal planes. All morphological images are from specimens collected at the type locality.

Adults in ethanol were chemically dried using hexamethyldisilazane. They were then pinned through the thorax with a minuten. The pupal exuviae and cocoon were placed in a glycerin microvial and pinned through its stopper beneath the associated adult. Any dissected parts (e.g., terminalia) were also included in a separate microvial and pinned beneath the relevant specimen. Descriptions of colors were taken from pinned specimens.

Morphological terminology follows that used by Adler et al. [21] and, for the larval mandible, Craig [25]. Morphological comparisons with all other members of the *S. annulus* group were made for larvae, pupae, males, and females, using descriptions and illustrations by Adler et al. [21] and Yankovsky [26].

### 2.4. Type Depositories

After larvae were prepared for chromosomal study, they were transferred from Carnoy’s fixative to 80% ethanol. The holotype and most paratypes were deposited in the United States National Museum (USNM), Washington, DC, USA. Additional paratypes (adults with pupal exuviae) were deposited in the Canadian National Collection (CNC), Ottawa.

### 2.5. COI Barcoding Procedures and Sequence Relationship Analysis

COI barcoding was performed on 8 larvae from the type locality. Larvae were fixed in 80% ethanol, which was replaced 3 times until it remained clear. Larval guts were removed to reduce potential contamination from other organisms. Mitochondrial genomic DNA (mtDNA) was extracted from individual larvae using the GeneJET Genomic DNA Purification Kit (Thermofisher Scientific, Waltham, MA, USA) according to the manufacturer’s recommended protocol. Extracted DNA was eluted in 50 μL elution buffer (10 mM Tris-Cl, pH 9.0, and 0.1 mM EDTA) and was used immediately or stored at −20 °C for polymerase chain reaction (PCR) experiments.

COI sequences were amplified using the standard LCO1490 and HCO2198 primer pair [27] in PCR reaction mix containing 10 mM of Tris-HCl, 50 mM of KCl, 2.5 mM of MgCl2, and 200 µM of each dNTP, 0.2 µM of each primer,1 U of AmpliTaq Gold^™^ DNA Polymerase (Thermofisher Scientific, Waltham, MA, USA), 4 µL of DNA, and an appropriate volume of purified H_2_O for a total volume of 25 µL. PCR cycling conditions were as follows: 1 min at 96 °C; 35 cycles at 95 °C for 45 s, 55 °C for 60 s, and 72 °C for 90 s; and 7 min of extension at 72 °C.

PCR products were visualized on 1.2% agarose gel stained by GelGreen dye (Biotium, Inc., Hayward, CA, USA). Bands of approximately 658 bp were excised and purified using the GeneJet Gel Extraction Kit (Thermofisher Scientific). Finally, approximately 10 µL (50 ng) of the 30-µL purified PCR products were mixed with 5 µL of 5 µM of either LCO1490 or HCO2198 primers for Sanger DNA Sequencing provided by Quintarabio, Hayward, CA, USA.

The raw sequencing reads of each specimen were analyzed in DNASTAR Lasergene to obtain consensus sequences, which were then examined and verified by performing a BLAST search in the GenBank^®^ nucleotide database and an identification search in the BOLD database. All sequences in this study were deposited in GenBank (accession numbers: OP256416–OP256423 for 8 sequences).

COI sequences with higher than 85% identity with known North America species in the BLAST search were selected for relationship analysis. Multiple sequence alignment using the MUSCLE algorithm and phylogenetic tree estimation using the Neighbor-Joining method [28] and Kimura 2-parameter model [29] were performed in MEGA 11 [30]. GenBank accession numbers for sequences used in the analysis were as follows: *Chironomus plumosus*: KX781763.1; *Parasimulium crosskeyi*: FJ524493.1; *Prosimulium mixtum*: KR515236.1; *Simulium aestivum*: FJ524569.1, FJ524570.1; *S. anatinum*: FJ524579.1; *S. annulus*: JF872769.1; *S. apricarium*: JF815152.1, JF815165.1, KJ662698.1, KM497540.1; *S. arcticum*: KJ662678.1, KJ662813.1, KM497546.1; *S. baffinense*: JF872771.1, FJ524582.1; *S. balteatum*: FJ524591.1; *S. bracteatum*: HM374133.1, MG158956.1; *S. brevicercum*: KJ662791.1, KM497568.1; *S. carbunculum*: FJ524632.1; *S. congareenaru*m: JF877007.1; *S. conicum*: FJ524638.1, FJ524639.1; *S. croxtoni*: FJ524654.1, KR671377.1; *S. curriei*: FJ524662.1, KR679937.1; *S. defoliarti*: JF868187.1; *S. emarginatum*: FJ524682.1; *S. excisum*: JF878006.1, KR753727.1; *S. exulatum*: JF877228.1, KR664724.1; *S. gouldingi*: FJ524701.1, HQ552022.1; *S. joculator*: FJ524722.1, FJ524728.1; *S. johannseni*: FJ524730.1, FJ524732.1; *S. maculatum*: NC 040120.1; *S. nebulosum*: KR444287.1, MG156442.1, MG159025.1; *S. pugetense*: FJ524763.1; *S. silvestre*: JF872847.1; *S. usovae*: JF872758.1; *S. ustulatum* n. sp.: OP256416; *S. violator*: KR646048.1.

## 3. Results

*Simulium ustulatum* Adler and Huang, n. sp.

### 3.1. Diagnosis

The larva of *S. ustulatum* n. sp. can be distinguished from that of all other simuliid species by the banded abdomen and the brownish triangular marking extended anteriorly from the apex of the postgenal cleft. The pupa is not reliably distinguished from those of other western species of the *S. annulus* group except *S. balteatum* Adler, Currie & Wood, which has elongated gill petioles. The male is unique among all simuliid species in having a combination of 3 long, stout distal parameral spines and a broad, flat ventral plate with a fingerlike lobe along its posterolateral margin. The female cannot be reliably distinguished on morphological grounds from those of other western species in the *S. annulus* group. Chromosomally, the combination of a massive chromocenter and unique fixed inversions in IS, IIS, IIL, and IIIL distinguishes *S. ustulatum* n. sp. from all other species of simuliids. 

### 3.2. Chromosomal Description

**Figure 2:**
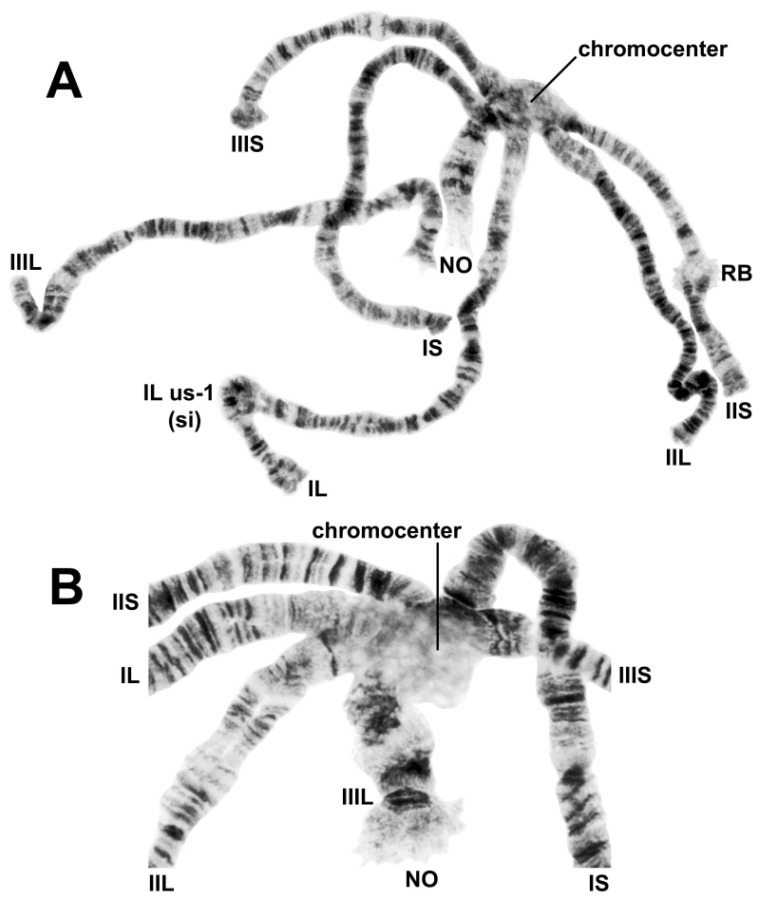
Polytene chromosome complement of *Simulium ustulatum* n. sp. (female larva), showing the 6 arms, chromocenter, and nucleolar organizer (NO). (**A**) Entire complement; RB, ring of Balbiani; IS us-1 (si), heterozygous configuration of inversion IS us-1. (**B**) Chromocenter and bases of all 6 arms.

The banding sequences of all 19 larvae (9 females, 10 males) prepared for analysis were fully interpreted. The chromosomal complement had a haploid number of 3 and exhibited standard arrangements and lengths for all 6 arms (Figure 2A). Synapsis of homologues, excluding heterozygous inversions, was tight, typically greater than 90%. The most conspicuous feature of the complement was a large chromocenter, which was typically pale-staining and somewhat vacuolated (Figure 2B). All arms radiated from the central hub and remained tightly attached to the chromocenter. The base of the IIIL arm proximal to and including the area of the nucleolar organizer was expanded (Figure 2B), and the base of IIIS was slightly expanded in some nuclei. The bases of the other 4 arms were not expanded.

**Figure 3:**
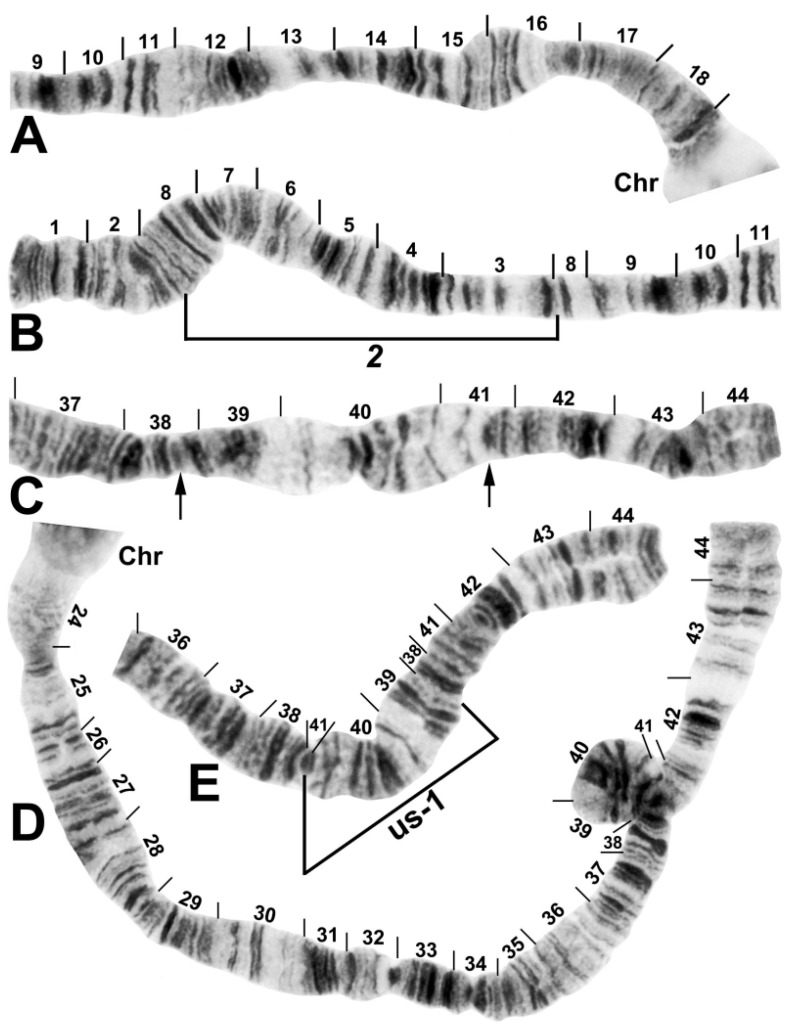
Chromosome I of *Simulium ustulatum* n. sp. (female larva). (**A**) Basal half of IS; Chr, chromocenter. (**B**) Distal half of IS showing the *IS-2* fixed sequence. (**C**) Distal half of IL, showing the standard sequence; arrows indicate breakpoints of autosomal inversion IL us-1. (**D**) Entire IL arm, showing heterozygous inversion IL us-1. (**E**) Distal half of IL, showing the homozygous inverted sequence for autosomal inversion IL us-1 (bracketed).

**Figure 4:**
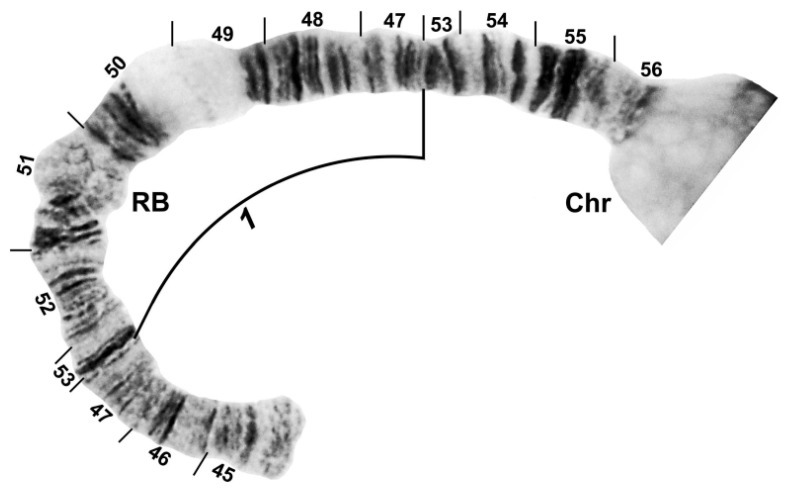
Chromosome arm IIS of *Simulium ustulatum* n. sp. (female larva), showing the *IIS-1* fixed sequence; Chr, chromocenter; RB, ring of Balbiani.

**Figure 5:**
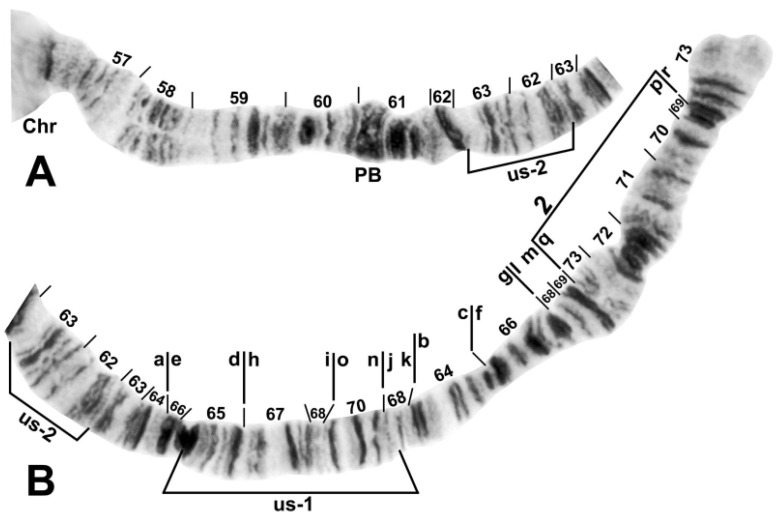
Chromosome arm IIL of *Simulium ustulatum* n. sp. (female larva). (**A**) Basal portion of arm, showing the homozygous IIL us-2 sequence; Chr, chromocenter; PB, parabalbiani marker. (**B**) Distal portion of arm, showing the complexly rearranged fixed sequence hypothesized to consist of 7 inversions, including the simple subterminal *IIL-2* inversion. Vertical bars above the chromosome indicate all fixed-inversion breakpoints. Alphabetizing the letters a–r produces the standard sequence for the *S. annulus* group. Two autosomal polymorphisms are indicated: The IIL us-2 sequence is present homozygously (bracketed), whereas the IIL us-1 sequence is not present, but its limits are bracketed.

**Figure 6:**
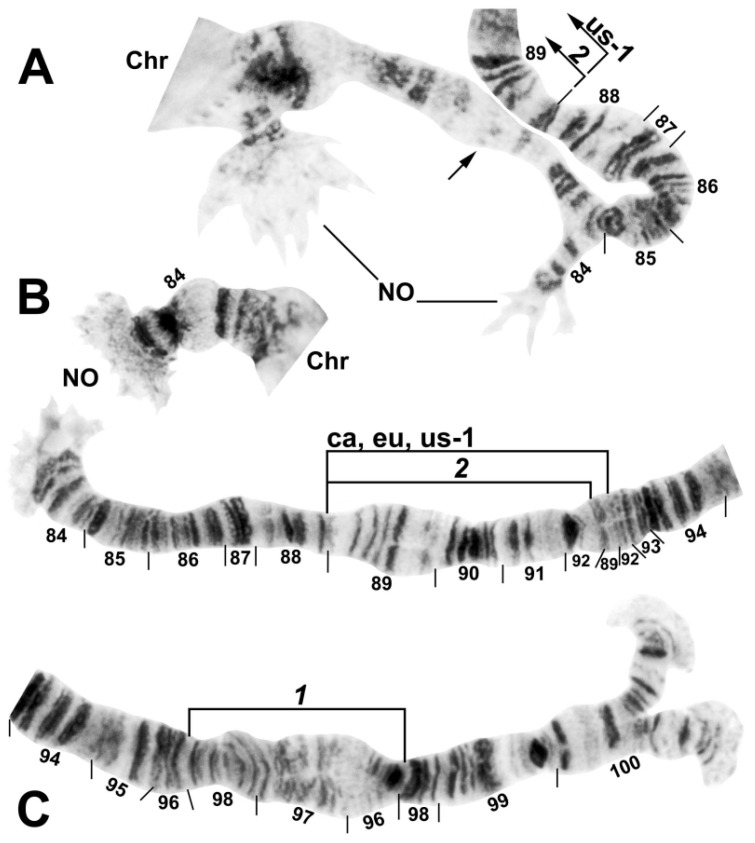
Chromosome arm IIIL of *Simulium ustulatum* n. sp. Chr, chromocenter; NO, nucleolar organizer. (**A**) Basal half of arm (male larva) showing the anucleolate Y, nucleolate X, and proximal portion of fixed inversion *IIIL-2* and polymorphic inversion IIIL ca, eu, em, Hi, us-1 (abbreviated as us-1); arrow indicates location of suppressed nucleolus on the Y. (**B**) Basal half of arm (female larva), showing the nucleolate XX condition and homozygous sequence for fixed inversion *IIIL-2* and polymorphic inversion IIIL ca, eu, em, Hi, us-1 (abbreviated as ca, eu, us-1). (**C**) Distal half of IIIL (female larva) showing the fixed *IIIL-1* sequence.

**Figure 7:**
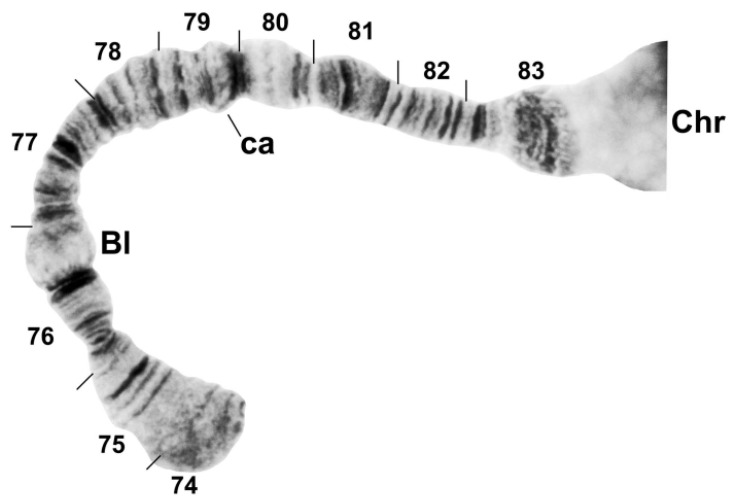
Chromosome arm IIIS of *Simulium ustulatum* n. sp. (female larva), showing the standard sequence for the *S. annulus* group. Bl, blister marker; ca, capsule marker; Chr, chromocenter.

The banding sequence of *S. ustulatum* n. sp. is removed from the group standard by 12 fixed rearrangements: *IS-1*, *IIS-1*, *IIL-1*, *IIL-2*, *IIL-3*, *IIL-complex* (consisting of 4 overlapping fixed inversions), *IIIL-1*, *IIIL-2*, and a transposed nucleolar organizer, albeit still within section 84 of the IIIL chromosome arm (Table 1, Figure 3, Figure 4, Figure 5 and Figure 6). *IS-1*, *IIL-2*, *IIL-complex*, and the transposed nucleolus (evidently without an inversion) are unique to *S. ustulatum* n. sp. *IIS-1* (Figure 4) is shared with *S. quadratum* (Stains & Knowlton) in which the inversion is linked to the Y chromosome [21]. *IIL-1* is shared with a number of other group members [22]. *IIL-3* and *IIIL-1* are equivalent to IIL eu-1 and IIIL eu-5, respectively, which are autosomal polymorphisms in *S. annulus* [22]; IIIL eu-5 is also shared with *S. quadratum* [21]. *IIIL-2* is shared with *S. balteatum* and *S. quadratum* [21]. IL (Figure 3C,D) and IIIS (Figure 7) had the standard banding sequence for the *S. annulus* group.

The IIL arm of *S. ustulatum* n. sp. is highly scrambled (Figure 5). The derivation of its fixed sequence from the group standard is hypothesized to have involved 7 inversion steps. *IIL-1* and *IIL-3* are assumed to have occurred first in the derivation from standard because they are shared with at least one other member of the *S. annulus* group. The letters *a* through *d* are assigned only as placeholders for each hypothesized inversion in *IIL-complex*. Several inversion sequences are possible, one of which is shown in the following derivation, where the letters a–r represent the sequence of chromosome fragments shown in Figure 5, slashes indicate breakpoints, and brackets indicate the inversion preceding the colon; the top sequence represents the new species:

*IIL-complex a*: a[ed/hi/on/jk/bc]fg/lm/qp/r
*IIL-complex b*: a[cb]kj/no/ih/defg/lm/qp/r
*IIL-complex c*: abc[kj/no/ih/defg]lm/qp/r
*IIL-complex d*: abc[gfed]hi/on/jklm/qp/r
*IIL-2*: abcdefghi/on/jklm[qp]r
*IIL-3*: abcdefghi/on[jklm]pqr
*IIL-1*: abcdefghi[onmlkj]pqr
Standard: abcdefghijklmnopqr

The sex chromosomes were based on IIIL (Figure 6). The X and Y were distinguished by nucleolar expression, with the Y chromosome being anucleolate. Thus, all females (Figure 6B) were homozygously positive for nucleolar expression (X_0_X_0_) and males (Figure 6A) were unexceptionally heterozygous (X_0_Y_1_).

Four autosomal polymorphisms were discovered (Table 1). IL us-1 (Figure 3C–E), IIL us-1, and IIL us-2 (Figure 5) were unique, and IIIL ca, eu, em, Hi, us-1 (Figure 6B) was shared with numerous members of the group. IIIL ca, eu, em, Hi, us-1 and *IIIL-2* are mimic inversions, differing by only a few bands (Figure 6B); hence, when both were present, the sequence superficially appeared to be the standard sequence. All but IIL us-1 were found in more than 50% of all homologues. IL us-1 was in Hardy–Weinberg equilibrium (Table 1), suggesting a panmictic population at the collection site. Larvae had a mean of 1.37 heterozygous autosomal inversions per larva.

### 3.3. Morphological Description

**Figure 8:**
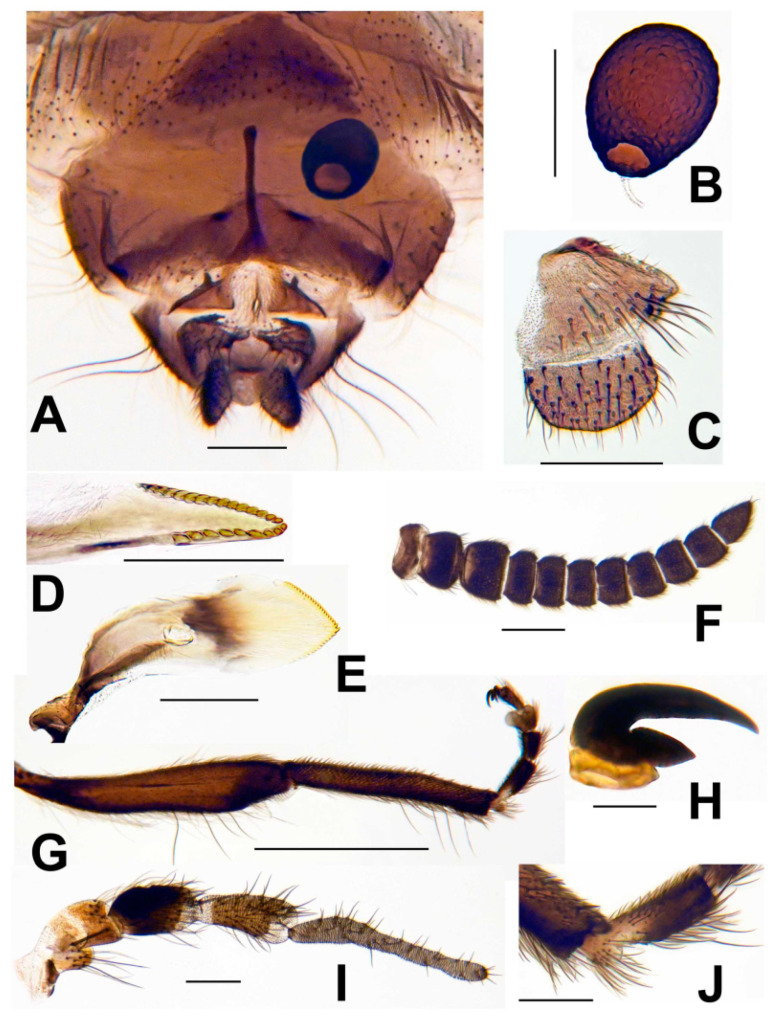
Female of *Simulium ustulatum* n. sp. (**A**) Terminalia and abdominal segments VII and VIII, ventral view. (**B**) Spermatheca and base of spermathecal duct. (**C**) Anal lobe and cercus, lateral view. (**D**) Lacinia, apex. (**E**) Mandible. (**F**) Antenna. (**G**) Hind tibia, tarsus, and acropod with claws. (**H**) Hind claw. (**I**) Maxillary palp, showing 5 palpomeres. (**J**) Apex of basitarsus showing the calcipala, and first tarsomere showing the pedisulcus. Scale bars: A–F, I and J = 0.1 mm; G = 0.5 mm; H = 0.02 mm.

**Female.** Thorax length 1.2–1.4 mm (mean = 1.3 mm, n = 9). Body grayish, pollinose, except lateral areas of abdomen velvety black; appendages dark brown, except halter off-white. All hair silvery, that of scutum with pale golden reflections. Head about 0.8 times as wide as thorax. Frons and clypeus with hairs primarily along margins; frons (narrowest diameter): head ratio 1.0: 6.0–7.0. Labrum about as long as clypeus. Antenna (Figure 8F) with scape, pedicel, and 9 flagellomeres; proportional lengths of pedicel, first flagellomere, and second flagellomere 1.5: 1.6: 1.0. Maxillary palp (Figure 8I) with proportional lengths of third, fourth, and fifth palpomeres 1.0: 1.1: 2.1–2.3; sensory vesicle ellipsoidal, 0.42–0.45 times length of third palpomere, with short neck and small mouth about 0.2–0.3 times length of vesicle. Lacinia (Figure 8D) with 23–25 teeth. Mandible (Figure 8E) with 14–17 inner and 23 or 24 outer teeth. Cibarium at junction with pharynx smooth, unarmed. Pleural membrane and postnotum bare. Katepisternum with minute patch of fine hair in some specimens. Precoxal bridge incomplete. Hind leg with basitarsus (Figure 8G) nearly parallel-sided, 7.8 times as long as wide, and 0.5 times as wide as greatest width of hind tibia; calcipala (Figure 8J) small, slightly shorter than wide, about 0.3 times as wide as apical width of basitarsus; pedisulcus (Figure 8J) well developed; claw (Figure 8H) with thumblike basal lobe. Wing 2.5–3.0 mm long (mean = 2.7 mm); subcosta dorsally and ventrally bare or with 1 or 2 hairs; radius with hair dorsobasally. Segment VII with well-sclerotized plate sternal plate. Ovipositor valves rounded posteromedially, membranous except inner margins weakly sclerotized; inner margins closely spaced, parallel. Genital fork (Figure 8A) with stem slender and well sclerotized; space between arms forming inverted U, and each arm expanded into large triangular lateral plate bearing prominent anteriorly directed apodeme. Anal lobe in ventral view (Figure 8A) with medially incised unpigmented area; anal lobe in lateral view (Figure 8C) produced ventrally as small lobe with unpigmented margin; anterior margin well sclerotized and pigmented. Cercus in lateral view (Figure 8C) short, rounded posteriorly, 1.9 times as wide as long. Spermatheca (Figure 8B) obovate, about 1.25 times as long as wide, heavily pigmented except broad area of juncture with duct unpigmented, with polygonal surface pattern; spermathecal duct and both accessory ducts unpigmented.

**Figure 9:**
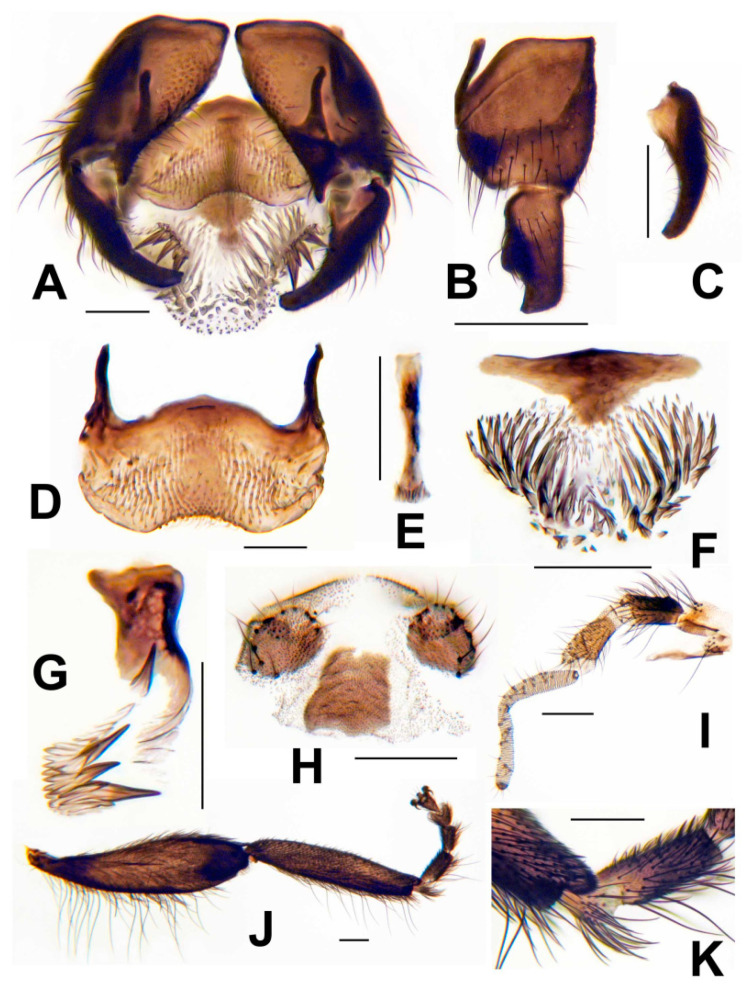
Male of *Simulium ustulatum* n. sp.; all scale bars = 0.1 mm. (**A**) Genitalia, ventral view. (**B**) Gonopod, lateral view. (**C**) Gonostylus, slight ventrolateral view. (**D**) Ventral plate, ventral view. (**E**) Median sclerite. (**F**) Dorsal plate and aedeagal membrane with spines. (**G**) Paramere and parameral spines. (**H**) Tergite X and cerci. (**I**) Maxillary palp, showing 5 palpomeres, and lacinia. (**J**) Hind tibia, tarsus, and acropod with claws. (**K**) Apex of basitarsus showing the calcipala, and first tarsomere showing the pedisulcus.

**Male.** Thorax length 1.0–1.2 mm (mean = 1.2 mm, n = 11). Body, antennae, palps, and legs uniformly dark brown, but scutum and abdominal dorsum and sides velvety black; halter grayish brown. All hair pale golden brown except those of stem vein and abdomen dark brown and brassy. Head as wide as thorax. Clypeus with hairs along lateral margins. Antenna with scape, pedicel, and 9 flagellomeres; proportional lengths of pedicel, first flagellomere, and second flagellomere 1.8: 1.8: 1.0. Maxillary palp (Figure 9I) with proportional lengths of third, fourth, and fifth palpomeres 1.0: 1.1: 2.2; third palpomere with sensory vesicle small, 0.15–0.17 times as long as third palpomere. Katepisternum, pleural membrane, and postnotum bare. Hind basitarsus (Figure 9J) somewhat parallel-sided, 5.2 times as long as its greatest width, 0.6 times as wide as greatest width of hind tibia; calcipala (Figure 9K) small, about as long as wide, slightly less than half as wide as apex of basitarsus; pedisulcus (Figure 9K) well developed. Wing 2.5–2.8 mm long (mean = 2.6 mm); radius with hair dorsobasally. Gonocoxite in ventral view about 1.2 times longer than gonostylus. Gonostylus in ventral and ventrolateral views (Figure 9A,C) 2.7–2.8 times as long as its greatest width, slender, smoothly curved toward midline, gradually tapered, with 1 apical spinule; in lateral view (Figure 9B) with basal 2/3 about 1.5 times wider than apical 1/3. Ventral plate in ventral view (Figure 9A,D) broad, flat, subrectangular, 1.8 times as wide as long, with finger-like lobe posterolaterally on each side; anterior margin convex; posterior margin broadly concave, covered with minute setae in broad triangular pattern; arms subparallel to each other; ventral plate in lateral view with sides subparallel and anterior margin projected toward apex of arms; ventral plate in terminal view rather flat, with lip barely produced. Median sclerite (Figure 9E) long, strap-like, doubled back slightly on itself at apex. Paramere (Figure 9G) with 1 stout spine near midlength and 3 stout spines, often with smaller spines, distally arising from mass of lobulate cuticle. Dorsal plate broadly subtriangular (Figure 9F). Aedeagal membrane (Figure 9A,F) on each side with about 30 slender spines and numerous additional smaller, shorter spines. Abdominal tergite X (Figure 9H) subquadrate. Cercus (Figure 9H) small, rounded, with 10 or 11 setae.

**Figure 10:**
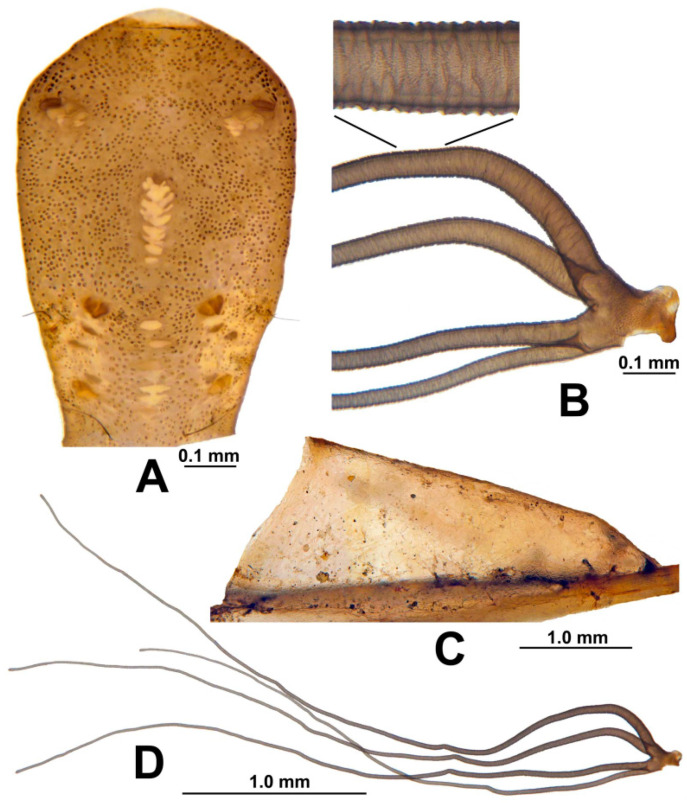
Pupa of *Simulium ustulatum* n. sp. (**A**) Cephalic plate of male, showing tubercles and trichomes. (**B**) Gill base with section magnified to show surface sculpture. (**C**) Cocoon affixed to stem of *Fontinalis hypnoides*. (**D**) Gill. Scale bars: A and B = 0.1 mm, C and D = 1.0 mm.

**Pupa**. Length (excluding gill) (n = 5) 2.9–3.8 mm, mean = 3.3 mm. Cephalic plate (Figure 10A) covered with minute, round tubercles; tubercles lacking on antennal sheaths; 1 long unbranched trichome near frons and 2–4 unbranched trichomes near antenna on each side. Thorax moderately covered with minute, round tubercles; 5 long, unbranched anterodorsal trichomes and 2 unbranched anterolateral trichomes immediately above leg sheaths on each side. Gill (Figure 10B,D) longer than pupa, 3.1–4.0 mm long (mean = 3.7 mm), with 4 slender, grayish, furrowed filaments in 2 pairs arising from short common basal stalk about as long as wide; stalks of dorsal and ventral pairs short, about as long as wide, with lower pair in dorsal view offset outwardly from vertical plane of dorsal pair by 25–35 degrees; dorsalmost filament arched basally and, along with other 3 filaments, directed anteroventrally; filaments decreasing in thickness from dorsal to ventral; filament surface with reticulate pattern (Figure 10B). Abdomen dorsally with segment I bearing 2 unbranched setae per side; segment II with 1 unbranched slender seta and 5 short setae per side; segments III and IV each with 4 recurved hooks and 0 or 1 unbranched, minute seta per side; segments V–VIII each with spine comb and 2 or 3 min setae per side; segment IX with anterior cluster of minute, stout microspines and pair of small, short terminal spines. Abdomen ventrally with segments III–VIII each with clusters of microspines; segments V–VII each with pair of stout, bifid or trifid hooks and 1 or 2 min, unbranched setae per side. Cocoon (Figure 10C) slipper-shaped, densely woven, yellowish brown; anterior margin slightly reinforced; length along dorsal midline (n = 9) 2.7–3.4 mm, mean = 3.1 mm.

**Figure 11:**
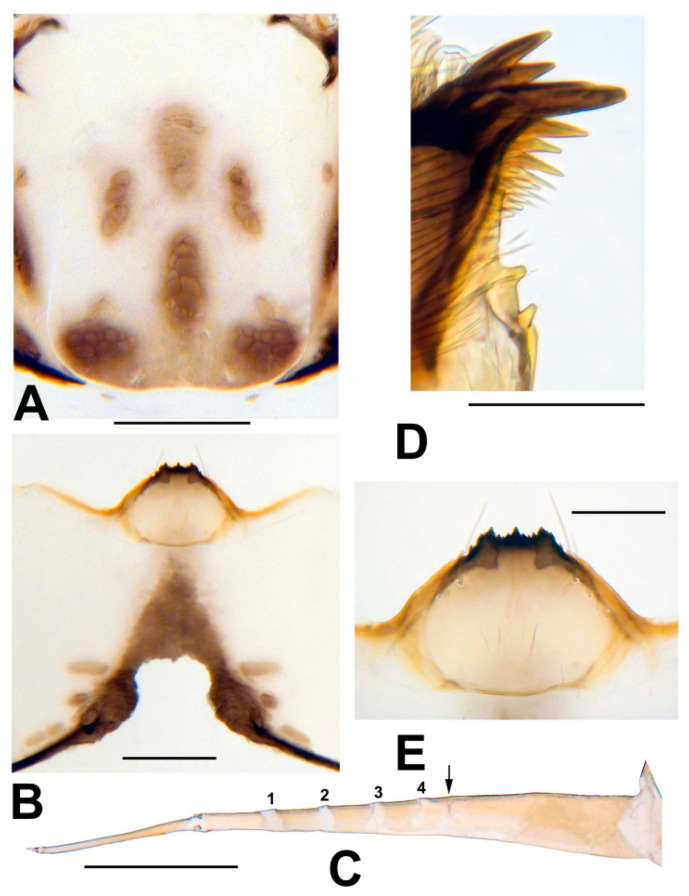
Larva of *Simulium ustulatum* n. sp. (**A**) Frontoclypeal apotome (**B**) Head capsule, ventral view. (**C**) Antenna; arrow indicates demarcation between proximal and medial articles and numbers 1–4 indicate the four hyaline bands of the medial article. (**D**) Apex of mandible. (**E**) Hypostoma. Scale bars: A and B = 0.2 mm, C and E = 0.1 mm, D = 0.05 mm.

**Mature larva**. Length (n = 9) 6.3–7.1 mm, mean = 6.7 mm. Body (in Carnoy’s fixative) cream-colored with burgundy band on each abdominal segment. Head capsule (Figure 11A) whitish yellow; head spots dark brown, well defined; sexual dimorphism of head pigmentation not apparent. Genae each with 2–4 faint, diffuse, brownish spots along posterior margin. Venter of head capsule (Figure 11B) whitish yellow, with brown, isosceles triangle extended toward hypostomal groove; horizontal long spot and round spot on each side of postgenal cleft brownish. Antenna (Figure 11C) pale yellowish brown, extended beyond apex of labral fan stalk by one-third to one-half length of distal article; medial article bearing 4 hyaline bands (sometimes requiring slide-mounting to see), plus additional hyaline band at junction of medial and proximal articles; proportional lengths of proximal, medial, and distal articles 1.0: 1.5: 1.1. Labral fan (n = 10) with 41–46, mean = 43.1 primary rays. Mandible (Figure 11D) with 3 outer teeth, 3 preapical teeth, numerous spinous teeth, and bifid mandibular sensilla; supernumerary serrations absent. Hypostoma (Figure 11E) with median and lateral teeth extended anteriorly to same level, with 2 or 3 paralateral teeth and 4–6 lateral serrations; 3 or 4 sublateral setae per side. Postgenal cleft (Figure 11B) short, somewhat quadrate, with rounded anterior corners, occasionally slightly biarctate, about 1/3 as long as postgenal bridge (measured from anterior margin of anterior tentorial pits to hypostomal groove). Cervical sclerites (Figure 11A) minute, free from occiput, widely separated from each other. Gill histoblast of 4 long, thread-like filaments. Abdominal cuticle (slide-mounted with stage diaphragm partially closed) with short, pale, unbranched setae. Rectal papillae of 3 finger-like lobes, each with 3 small, short lobules. Anal sclerite X-shaped, with anterior arms about as long as posterior arms, thinner, and paler. Last abdominal segment with pair of large conical ventral tubercles. Posterior circlet with 66–69 rows of 9–13 hooklets per row.

### 3.4. Type material

Holotype (USNM): Male (pinned) with pupal exuviae and cocoon (in associated glycerin vial), California, San Joaquin County, Lower Mokelumne River, 38°12′36″ N 121°22′32″ W, 4.5 m asl, pupa collected 21 March 2022 by S. Huang. Paratypes (USNM and CNC): Same location and collector as holotype, 28 larvae (19 with posterior excised and chromosomally stained, 4 pharate pupae, and 5 middle instars; transferred from Carnoy’s to 80% ethanol), 8 March 2022; 11 pupae (in 80% ethanol), 21 March 2022; 10 males and 9 females (each pinned with pupal exuviae and cocoon in associated glycerin vial), 21 March 2022.

### 3.5. Other Specimens Examined

Twelve larvae (in 80% ethanol), California, San Joaquin County, Stanislaus River, 37°42′10″ N 121°10′39″ W, 9 m asl, 7 March 2022, collected by S. Huang.

### 3.6. Bionomics

Other species of black flies collected with *S. ustulatum* n. sp. in the Lower Mokelumne River included 1 larva of *Simulium donovani* Vargas, more than 50 larvae and 2 pupae of *Simulium vittatum* Zetterstedt, five larvae of *Simulium clarum* (Dyar & Shannon), and 1 pupa of the *Simulium tuberosum* species complex. In the Stanislaus River, *Simulium clarum* was the predominant species. Like all other members of the group, *S. ustulatum* n. sp. is probably univoltine. Ornithophilic feeding habits are inferred from the bifid claws of the females and the feeding habits of related species [21].

### 3.7. Etymology

The species name *ustulatum* is from Latin, meaning singed or browned, in reference to the diagnostic brownish triangular marking on the larval head capsule at the apex of the postgenal cleft.

### 3.8. Morphological Comparisons with Other Species

Among Nearctic species, the female of *S. ustulatum* n. sp. resembles all other western North American members of the *S. annulus* group. The male is most similar to that of *S. balteatum* by being the only species with three stout distal parameral spines. Like *S. annulus*, *S. ustulatum* n. sp. has a finger-like lobe posterolaterally on each side of the male ventral plate, which is expressed as a result of the posterolateral margin curling slightly upward. The three distal parameral spines and the bulged basal two-thirds of the gonostylus, however, distinguish *S. ustulatum* n. sp. from *S. annulus*. The pupa resembles that of *S. annulus*, *S. canonicolum*, *S. quadratum*, and *S. zephyrus*. The larva is most similar to that of *S. canonicolum*, *S. quadratum*, and *S. zephyrus*, but has the brownish triangular marking on the ventral head capsule.

About 13% of the simuliid species known from the Nearctic Region are Holarctic, although the species shared between the Nearctic and Palearctic are largely northern in distribution; only about 1% of species south of 40 °N are shared [21,31]. We, therefore, morphologically compared *S. ustulatum* n. sp. with the four species known from the Palearctic Region, none of which has been examined chromosomally. *Simulium annae* (Rubtsov), known only from the female and pupa, and *S. olonicum* (Usova) have rarely been reported beyond their type localities in northwestern Russia. The absence of a conspicuous anteriorly directed apodeme on each lateral arm of the genital fork of *S. annae* distinguishes it from *S. ustulatum* n. sp. The morphological similarity of *S. olonicum* to *S. annulus* suggests possible conspecificity; the lack of a pattern on the ventral head capsule of the larva distinguishes *S. olonicum* from *S. ustulatum* n. sp. The absence of antennal annulations and lack of a brown triangular pattern on the ventral head capsule of the larva, and the distal branching of the lower pair of gill filaments, are among the characters that distinguish *S. tokachiense* Takaoka, Otsuka & Fukuda, a Japanese species, from *S. ustulatum* n. sp. [32]. The presence of multi-branched abdominal setae and lack of a brown triangular pattern on the ventral head capsule of the larva, and the cibarial tubercles of the female, are among the features that distinguish the Far Eastern species *S. konoi* (Takahasi) from *S. ustulatum* n. sp. [33].

### 3.9. COI Barcoding

**Figure 12:**
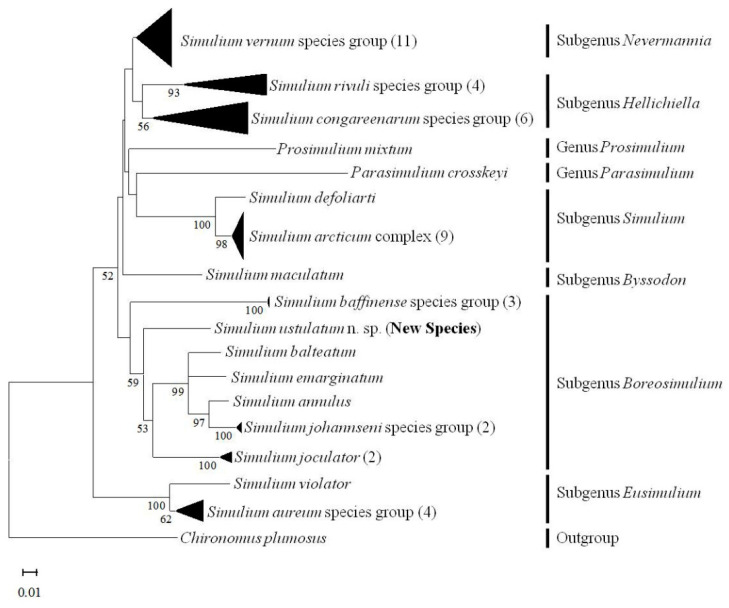
MtDNA tree inferred using the Neighbor-Joining method and Kimura 2-parameter model. The original optimal tree is shown. The bootstrap was set at 1000 replicates [35]. The percentage of replicate trees higher than 50% in which the associated taxa clustered together in the bootstrap test are shown below the branches. The tree is drawn to scale, with branch lengths in the same units as those of the evolutionary distances used to infer the tree. Branches clustered with taxa from the same groups or complexes are collapsed, and the numbers of taxa or sequences are shown in parentheses after the names of the species group or species complex. The length and height of the right edges of the black triangles are proportional to the number of taxa and to the range of distance in the branch, respectively.

Eight COI sequences of 658-bp length were identified for *S. ustulatum* n. sp. A GenBank BLAST search yielded 38 sequence hits with greater than 90% sequence identity (Table 2). Members in the *S. annulus* species group had higher sequence identity with *S. ustulatum* n. sp. than did other known species. The highest identity was with *S. balteatum*, although we note that two western group members (*S. quadratum* and *S. zephyrus*) are not in GenBank or BOLD. Two Palearctic species in the subgenus *Nevermannia*, *Simulium lundstromi* (Enderlein) and *Simulium angustitarse* (Lundström), shared higher sequence identity with *S. ustulatum* n. sp. than did the *Simulium baffinense* species group, which is currently in the same subgenus (*Boreosimulium*) as the *S. annulus* and *S. johannseni* species groups. Additional species showing sequence identity greater than 90% included unidentified taxa and *S. maculatum* (Meigen). An identification search in the BOLD database generated nearly identical results showing that the COI sequences for *S. ustulatum* n. sp. share higher identity (about 92%) with members of the *Simulium annulus* group than with any other taxon in the BOLD database. The maximum intraspecific divergence of COI sequences in a previous study of Nearctic species is below 3.84% [34], suggesting that the COI sequences obtained in this study do not belong to any species currently in GenBank or BOLD. The tree generated by using the Neighbor-Joining method and Kimura 2-parameter model revealed strong support for a relationship between *S. ustulatum* n. sp and other members of the *S. annulus* species group (Figure 12).

## 4. Discussion

### 4.1. Chromosomes as Barcodes

Polytene chromosomes represent physical maps, or barcodes, of the simuliid macrogenome and are replete with information about species limits, degree of reproductive isolation, population genetics, and speciation. Although operating at different genetic scales, the traditional DNA barcode of only one gene or part of one gene (typically COI) samples the microgenome, whereas the chromosomal barcode samples the entire macrogenome. Compared to an organism’s morphology, the chromosomes also offer a more complete set of characters. The vast number of structural features, particularly when all life stages are considered, can be overwhelming, and taxonomists tend to focus on a small subset of structural features, typically those that have historically been used in the group under study. The greatest practical difference between polytene chromosomes and genes as barcodes is the effort required for interpretation. Although chromosomal preparation is simple, typically using either Feulgen or orcein staining techniques [21,36], interpretation of banding sequences and other chromosomal phenomena requires training, practice, and patience. 

This comparison is not to claim that one barcode is better than the other. Rather, if these tools are used appropriately [24,37], both have value, especially when coupled with morphology. Information extracted from chromosomes is not necessarily available at another level such as the gene or the organism. The COI gene, for example does not always distinguish cryptic species of simuliids even when polytenes demonstrate reproductive isolation in sympatry [38]. In limited cases, polytenes do not provide resolution of putative species when the COI gene suggests their presence [39]. Homosequential species, those species not differing in their fixed sequences of chromosomal bands but differing significantly in their morphology, are also represented among simuliids [21]. More problematic are homosequential cryptic species, which do not differ in their fixed chromosomal band sequences or in their morphology [40,41]. They are typically recognized by linkage disequilibrium of autosomal polymorphisms in the chromosomes. Molecular tools have not been brought to bear on homosequential cryptic species in the Simuliidae. Most challenging are putative species that do not differ in their chromosomes, COI barcodes, or morphology [42] but that might have other genic differences or show developmental, ecological, or behavioral incongruencies. 

The purely taxonomic value of using multiple approaches lies in the potential to discover and identify all life stages and both sexes. The utility of polytene chromosomes is typically limited to the antepenultimate- to ultimate-instar larvae. The structural uniformity of certain life stages, particularly the egg, of black flies disables morphological identification. In the *S. annulus* group, the structural homogeneity of the females and the pupae of some species renders the DNA (COI) barcode invaluable for identification. The inability to identify female black flies to species can be costly, given that females cause the pest problems and transmit disease organisms. The use of multiple approaches also guards against problems of misidentification. GenBank includes misidentifications of black flies, and we strongly suspect that the specimens of *S. johannseni* in GenBank represent misidentifications, probably of *S. annulus*, with which they cluster in our study and in the original study that identified the specimens as *S. johannseni* [34]. The inarguable conclusion is that integrated taxonomic approaches, including the use of multiple genes, are the most profitable.

### 4.2. Chromosomal Similarities and Novelties

*Simulium ustulatum* n. sp. is only the second species of black fly described from North America in the past 18 years. The chromosomal barcode indicates its species status, and the morphological features reinforce its specific distinction. It differs chromosomally in far more respects from the *S. annulus* group standard than do any of the other Nearctic group members. Yet, morphologically, it is consistent with all other species in the *S. annulus* group and is no more distinct than other members of the group. Not all cytological features given originally [22] to indicate cohesiveness of the group apply to *S. ustulatum* n. sp. Namely, the centromere region of chromosome I is not expanded, the standard sequence does not occur in the IIS or IIIL arm, and there is no scarcity of fixed-inversion differences from other species in the group. On the contrary, *S. ustulatum* n. sp. has the greatest number of fixed rearrangements (twelve) relative to the other eight chromosomally studied members of the group, which have only one fixed rearrangement or none.

The tendency for the centromeres of the three chromosomes to pair ectopically in species of the *S. annulus* group in the Nearctic Region ranges from frequent [21,22] to negligible [23]. Those species that express frequent ectopic pairing of centromeres (i.e., a pseudochromocenter), however, lack the heterochromatic mass that constitutes the true chromocenter seen in *S. ustulatum* n. sp. The evolution from a non-chromocentric to fully chromocentric state might occur via ectopic pairing of the centromeres, with progressive addition of heterochromatin [43,44]. Ectopic pairing can be loose or tight and involve some or all nuclei in an individual larva [45]. If chromocenters evolve from the simplest centric associations, such as loose ectopic pairing, the implication is that chromocentric forms within a taxon are more derived. Heterochromatinization has been identified as a driving force in speciation [46]. We note one other aspect of simuliid chromocenters: the intensity of their Feulgen-staining. True chromocenters in the Simuliidae are either pale and glassy, as in some *Prosimulium* species [45] and *S. ustulatum* n. sp., or darkly staining as in *S. chromatinum* Adler, Currie & Wood and *S. chromocentrum* Adler, Currie & Wood [21]. No phylogenetic trend is apparent and might simply be related to the amount of heterochromatin.

*Simulium ustulatum* n. sp. shares one to six rearrangements with other species in the group. An anucleolate Y, for example, is common in the group, occurring in four of the nine Nearctic species, including *S. ustulatum* n. sp. [21,22,23]. Six rearrangements are shared with *S. annulus* (anucleolate Y; *IIL-1*; IIL eu-1 [= *IIL-3*]; IIIL eu-5 [= *IIIL-1*]; IIIL ca, eu, em, Hi, us-1; and *IIIL-2* which is Y-linked in some eastern populations (P.H.A. unpublished data). Four rearrangements are shared with *S. quadratum* (IIS-1, *IIL-1*, IIIL eu-5 [= *IIIL-1*], and *IIIL-2*) and three with *S. balteatum* (anucleolate Y, *IIL-1*, and *IIIL-2*). Although shared chromosomal features suggest a sister-group relationship with one, two, or all three of these species, the original arbitrary selection of the standard sequence for the *S. annulus* group [22] precludes a cytophylogeny. In other words, determining whether the various chromosomal rearrangements are synapomorphic or plesiomorphic is not possible without outgroup comparison. The significantly rearranged complement of the *S. annulus* group, relative to other species groups, does not allow a comparison with outgroups at this time.

### 4.3. Implications of Discovery of the New Species

*Simulium ustulatum* n. sp. is geographically nearest to *S. balteatum*, *S. joculator* Adler, Currie & Wood, and *S. quadratum*. It is, however, the only member of the *S. annulus* group in western North America that has been collected near sea level (4–9 m asl). The vast majority of western collections of the group have been made in the mountains. The early development of *S. ustulatum* n. sp. ensures the requisite flow of cold water in which all members of the group develop. Global climate change (e.g., increasing temperatures and smaller snowpacks), impoundment, diversion of flow (e.g., for irrigation and municipal use), and increasing agricultural and land development in California’s Central Valley threaten the species if it is confined to low-elevation rivers.

## Figures and Tables

**Table 1:** Frequency of rearranged chromosomal homologues in larvae of *Simulium ustulatum* n. sp. (n = 9 females, 10 males) from the type locality (Mokelumne River, California, 8 March 2022), relative to the standard banding sequence of the *Simulium annulus* species group.

Rearrangement	Frequency
*IS-2*	1.00
IL us-1	0.58 ^1^
*IIS-1*	1.00
*IIL-1*	1.00
*IIL-2*	1.00
*IIL eu, us-1*	1.00
*IIL-complex* ^2^	1.00
IIL us-1	0.10
IIL us-2	0.74
*IIIL-1*	1.00
*IIIL-2*	1.00
IIIL ca, eu, em, Hi, us-1	0.71
IIIL N.O. transposition	1.00
IIIL N.O.	* ^3^

^1^ IL us-1 was in Hardy–Weinberg equilibrium (df = 1, χ^2^ = 2.3578, *p* > 0.1247): ss = 5, si = 6, ii = 8, where s = standard and i = inverted. ^2^ The derivation of the IIL arm of *S. ustulatum* n. sp. from the standard chromosome sequence for the *S. annulus* group involves multiple fixed inversions, here designated *IIL-complex* and hypothesized to consist of 4 inversions. ^3^ * = Expression of the nucleolar organizer (N.O.) was sex-linked: X = nucleolate, Y = anucleolate.

**Table 2:** Sequences most similar to *Simulium ustulatum* n. sp. in a GenBank BLAST search.

Species	Number of Hits *	Percent Identity
*Simulium balteatum*	5	91.7–92.5
*Simulium emarginatum*	7	91.4–92.4
*Simulium* sp. BIOUG24287-A03	1	92.20
*Simulium* sp. BIOUG24289-E02	1	92.20
*Simulium annulus*	4	91.2–91.5
*Simulium johannseni*	4	91.1–91.4
*Simulium lundstromi*	9	90.1–90.7
*Simulium angustitarse*	3	90.3–90.6
Simuliidae sp. BOLD:AAG9573	3	90.1–90.6
*Simulium maculatum*	1	90.30

* Initial BLAST search with default settings generating more than 5000 hits. These hits were then filtered to percent identity and query coverage, both at 90–100%.

## Data Availability

All data supporting reported results are included in the text.
